# Role of RNA in genome folding: It’s all about affinity

**DOI:** 10.1016/j.sbi.2025.103136

**Published:** 2025-08-20

**Authors:** Rafal Czapiewski, Nick Gilbert

**Affiliations:** MRC Human Genetics Unit, Institute of Genetics and Cancer, The University of Edinburgh, Crewe Rd, Edinburgh, EH4 2XU, UK

## Abstract

In mammalian cells, RNA species make up ~10% of chromatin by mass and play a structural role in the nucleus by acting as scaffolds and influencing genome organisation. Although many proteins bind nuclear RNAs, these interactions are often non-specific, making it challenging to define RNA’s role in genome folding. Nonetheless, a clearer picture is emerging. Some RNAs, like NEAT1 and MALAT1, have high affinity for specific RNA-binding proteins and form the basis for nuclear bodies. In contrast, many nuclear proteins bind RNA weakly, resulting in numerous low-affinity interactions. We propose that these interactions generate a complex RNA-protein network with dynamic, gel-like properties that modulate chromatin folding and transcription factor mobility. This suggests an exciting feedback mechanism in which newly transcribed RNA contributes directly to shaping chromatin architecture.

## Introduction

In higher eukaryotes chromatin is composed of approximately equal amounts by mass of DNA and protein, but researchers often neglect the contribution of RNA [1]. One possible reason is that RNA has multiple roles in cells. It functions as the information conduit between DNA and proteins, but additionally, RNA molecules can fold into different shapes that exhibit catalytic activity or behave as scaffolds to facilitate other cellular processes. We have known that RNA is a component of chromatin since the 1970s, but its function is rather ethereal and difficult to ascribe, not least the origin of RNA in the nucleus is extremely diverse [2]: derived from coding and non-coding genes, processed or unprocessed transcripts, or as lariats, the discarded by-products of RNA splicing. Acknowledging the importance of nuclear RNA species, researchers have been developing new experimental approaches to investigate the origin and life story of these RNAs, and to better understand how they play a role in regulating critical nuclear functions such as in genome folding. In this review we will focus on the role of RNA in regulating chromatin and genome folding, in particular, focusing on the importance of the biophysical properties of RNA. One particularly unique nuclear RNA is *XIST*, a 17 kb long RNA molecule that is required for transcriptionally silencing one copy of the two X chromosomes in human females. Due to space limitations, we will not discuss the role of *XIST* in gene silencing and genome folding, but instead refer readers to other recent reviews [3,4].

## Biophysical behaviour of chromatin-associated RNA

**Figure 1:**
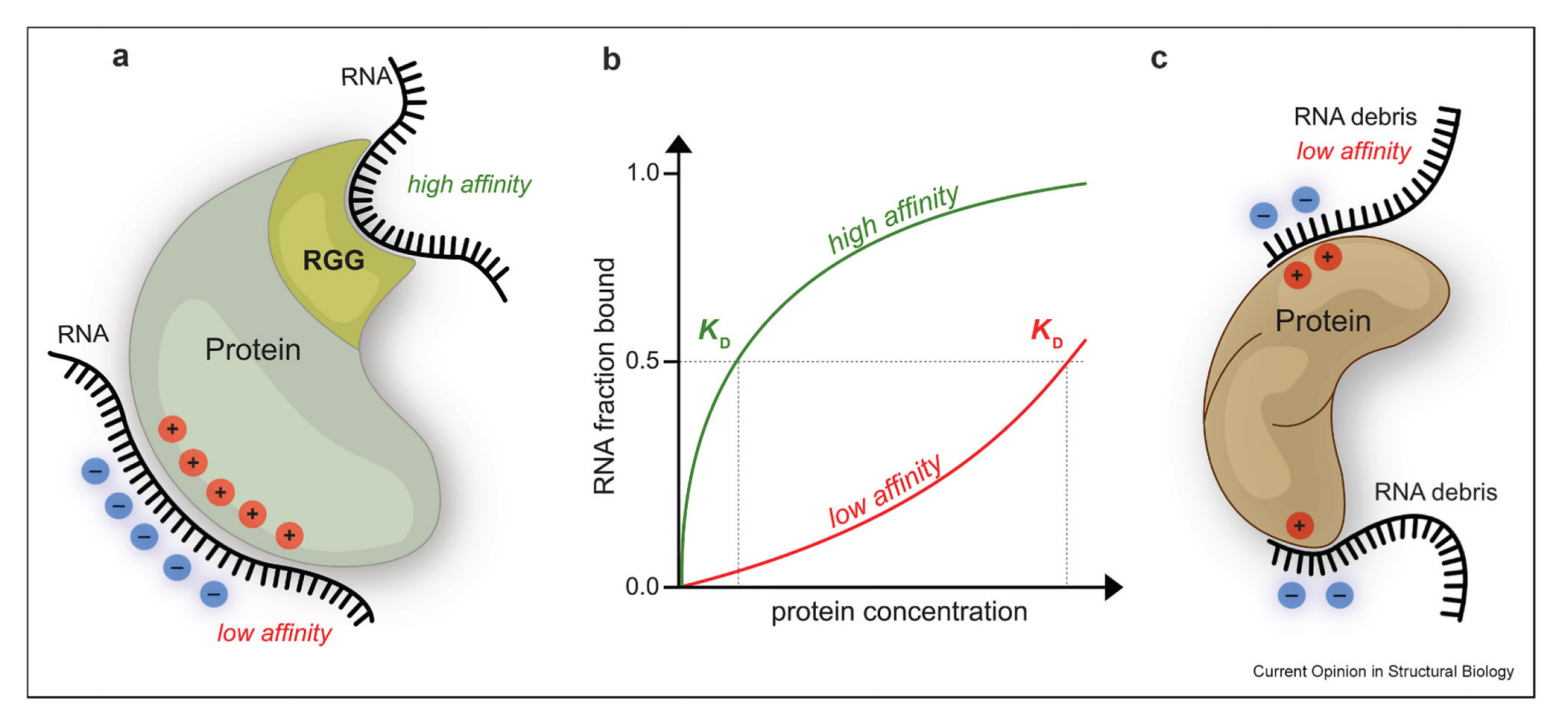
Different modes of protein/RNA binding can have different affinities. **(a)**. Interactions based on electrostatic charge often have lower affinity and specificity. The inherent negative charge of RNA molecules drives weak interactions with positively charged proteins or protein domains. RGG domains, however, are recognised for having higher affinity and specificity. In addition to the electrostatic interactions driven by the positively charged arginines in RGG domains, there are also other multivalent bonds with the RNA, such as hydrogen bonds or π-stacking [8]. **(b)**. These differences in RNA-protein affinity can be verified experimentally through a saturation curve assay and the dissociation constant *K*_D_ calculation. Here, a theoretical plot is shown where high-affinity RNA-binding proteins exhibit lower *K*_D_ values, while the opposite is true for low-affinity binding. **(c)**. A protein with a low affinity for RNA binding.

Studies examining a role for RNA are confounded by it being inherently sticky, it has an ability to base pair (A with U, G with C) with itself or with other RNA molecules, leading to many different secondary structures and interactions [5]. As it is strongly negatively charged it can of course interact with many protein species both non-specifically and through canonical RNA-binging domains, which are often arginine-rich [6—8]. Typically, specific interactions are highly targeted, often occurring between defined RNA species and canonical RNA-binding domains, whilst non-specific interactions are more generic, However, it is difficult to discriminate between these different modes of binding [7], and it has been reported that RNA-binding proteins that lack canonical RNA-binding domains are rarely sequence-specific [9] (Figure 1). It should also be noted that some proteins have both DNA and RNA binding activities [10], one being SAF-A (scaffold attachment factor A) or HNRNPU (heterogenous ribonucleoprotein U), which we discuss later. Across the human genome over 400 proteins contain well-studied RNA-binding domains [11]. In contrast assays to examine the RNA interactome suggest that thousands of proteins crosslink to both coding and non-coding RNAs [12,13]. Many of these, however, do not have a reported role in RNA-binding, regulation or metabolism but could be moonlighting as weak RNA interactors [14,15]. This, however, maybe moot, as it is quite possible that RNA can play important roles through both these different binding modes. Instead, the effect of RNA on nuclear function is mediated either by RNA-binding proteins [16] or by competing with protein—protein interactions [17], which may affect liquid—liquid phase separation [18].

**Figure 2:**
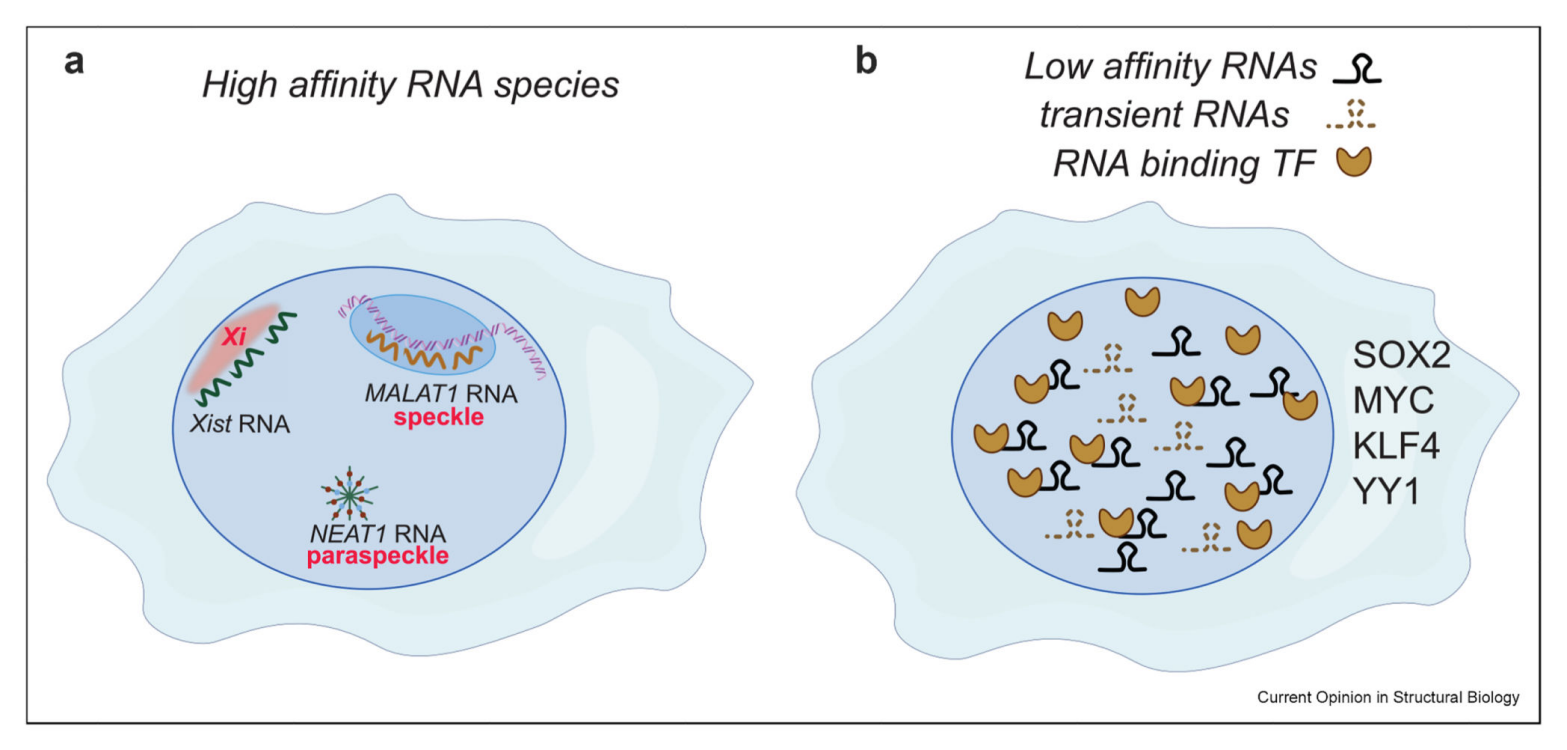
RNA-dependent nuclear bodies. Nuclear localisation and distribution of RNA and protein complexes depend on binding affinity. **(a)**. RNA species, such as *XIST, NEAT1* or *MALAT1*, bind to proteins with high affinity and exhibit clustered localisation, often in RNA-dependent structures within the nucleus, such as the inactive X chromosome, paraspeckles or speckles. **(b)**. Lowaffinity complexes, like nascent RNA and transcription factor interactions (e.g. SOX2, MYC, KLF4 or YY1), show diffused localisation, featuring a mix of bound and unbound species.

When considering how RNA might affect the structure of chromatin it is important to think of the different classes of RNA that might be found in the nucleus [5]. The best understood classification of RNAs is the separation into coding and long non-coding RNAs (lncRNAs), which are generally synthesised by RNA polymerase II [19], and attached to chromatin via the polymerase during transcription. Surprisingly, there are 18,000 lncRNA genes in humans, with the majority being capped, spliced and polyadenylated, and if they are exported to the cytoplasm, they are packaged with RNA-binding proteins, to form mRNPs [20]. Although there will be exceptions, it seems that a small class of lncRNAs such as XIST, NEAT1 or MALAT1, play a scaffolding role in the nucleus to form nuclear bodies (Figure 2a; see later), whilst the remainder can be thought of as structure/function managers in the nucleus or cytoplasm, but with more diffuse roles (Figure 2B). In contrast nascent RNAs (derived from coding genes, or repetitive elements) and RNA by-products of splicing seem to act as transient bulking agents in the nucleus [21,22]. Some researchers have referred to these nuclear RNAs as being chromatin-associated or caRNA (chromatin-associated RNA). This terminology implies there are specific interactions between the RNAs and the chromatin fibre, but we now think that many of these interactions are relatively weak non-specific associations. The term caRNA is a catch-all but is potentially too general. Instead, we propose emphasising the affinity of RNA binding to nuclear structures and instead talk of ‘chromatin-bound RNAs’ for high-affinity interactions, and ‘RNA debris’ [5] for low-affinity interactions. This terminology will of course be contentious, but might be a useful discussion point. We also need to use and develop approaches to discriminate between these modes of binding, and suggest that more detailed analyses of RNA binding and studying turnover kinetics could provide additional insights (see later).

## New approaches for characterising chromatin-associated RNAs

New techniques are being developed for examining the panoply and kinetics of different RNA species in the nucleus. Studies using nanopore sequencing suggest that a whole variety of different RNAs are produced [23,24], with RNA processing kinetics varying between genes [25]. In TimeLapse-seq, kinetic labelling of RNA using 4sU enabled the kinetics of RNA flow through the nucleus to be measured, particularly nuclear export and RNA degradation [26]. Interestingly nuclear RNA half-lives are very closely correlated with chromatin half-lives, export is relatively rapid (<15 min), and most RNA in the nucleus is closely associated with chromatin with half-lives of 21—170 min. But what do we mean by RNA interaction with chromatin? Actively transcribing RNAs will be attached to the chromatin fibre by RNA polymerase II, so an average 30 kb gene (assuming transcription at 2 kb/min) will be attached for 15 min. From Ref. [26], it then appears that RNAs may remain in the vicinity of the chromatin fibre for a further 30 min. As RNAs do not diffuse rapidly [27,28], we suggest that instead they can become transiently associated with an RNA/protein mesh [21,22,28] (and see later). From our experiments [22] and those of others [21], it appears that the RNAs transiently associating with chromatin are a mixed population of different RNA species [5]. Some of these RNAs might be waiting to be processed, others derived from spliced-out introns, abortive transcripts, or repetitive C_0_T1 RNAs that are derived from transcribed repetitive elements such as LINES and SINES [21].

Multiple approaches have been developed over the last few years for comprehensively mapping RNA binding to chromatin [29—31]. Most of these techniques rely on linking RNA fragments to the DNA, so that when the hybrid polynucleotides are sequenced, the RNA can be linked to a genomic location. Interestingly many of these studies suggest that RNAs stay in close proximity with the genomic region from which they are transcribed. This suggests that there is relatively little diffusion of RNA in the nucleus, but also argues that there is little evidence for RNA species acting in trans over long distances [30].

A different approach for examining RNA-DNA interactions in the nucleus is called RD-SPRITE (RNA & DNA SPRITE). Unlike other methods, RD-SPRITE does not rely on proximity ligation and instead uses a split-pool approach to identify which RNA and DNA fragments are in close proximity to each other. This approach demonstrated that chromatin and RNA are closely associated in 3D space, particularly in the context of RNA processing, heterochromatin assembly and gene regulation [32]. By extending this method and defining a parameter called the SPRITE speckle proximity score, the relationship between different genomic regions and speckles could be examined [33]. Excitingly this showed that the genes localised near nuclear speckles have higher co-transcriptional splicing, but also that recruitment of pre-mRNA to speckles can increase splicing levels or efficiency. These results are complimented by a study from the Henikoff lab where they evolved their CUT&TAG technology for mapping RNA. In CUT&TAG (Cleavage Under Targets and Tagmentation), the Tn5 transposase is recruited to specific regions of the genomic using an antibody to the required target. Activation of the transposase using magnesium ions generates fragments for next-generation sequencing.

The authors have modified this approach in two ways, firstly in a revised method called Reverse Transcribe and Tagment (RT&Tag), a localised reverse transcription reaction generates RNA/cDNA hybrids that take advantage of Tn5 transposase’s activity on RNA/DNA hybrids, which are tagmented by the transposase for subsequent sequencing [34]. In the latest iteration RT&Tag has been combined with 4sU labelling to reveal of RNA processing [35]. Using this approach they suggested that one role for nuclear speckles is to trap incompletely spliced transcripts and facilitate their complete processing. In addition to new experimental approaches for identifying RNA-chromatin interactions, researchers have also started to build new computational pipelines. For example, the AkitaR package integrates RNA-DNA interactions with the genome to understand chromatin-associated RNAs, and in particular their cis- and trans-regulatory roles [36].

## RNA is a scaffold for nuclear bodies

Many nuclear bodies―such as speckles, paraspeckles and polycomb bodies―depend on RNA species for their formation and maintenance [37,38]. These nuclear bodies are often formed via macromolecular phase separation, a process that can vary greatly in size depending on the local concentration of factors. Phase separation is driven by interactions between oppositely charged molecules, including RNA and proteins. In the case of splicing speckles, key nuclear domains involved are the serine/ arginine-rich (RS) domains of scaffold proteins such as SRRM2 and SON. These non-redundant proteins create specialised microenvironments that facilitate RNA processing via proteins like SRSF1, and the splicing factor condensates they form can themselves regulate RNA processing and impact specific cellular processes such as hypoxia-induced alternative splicing [39].

Well-characterised RNAs that act to broadly form nuclear bodies are XIST, MALAT1 (Metastasis Associated Lung Adenocarcinoma Transcript; 8.7 kb in humans) and NEAT1 (nuclear paraspeckle assembly transcript; 3.2 kb in humans). MALAT1 and NEAT1 are highly transcribed and, by RNA FISH, are co-localised with splicing speckles and paraspeckles, respectively. NEAT1 is crucial for paraspeckle assembly, and in its absence paraspeckles disintegrate [40], whilst depletion of MALAT1 has little effect on the formation of splicing speckles [41]. It appears that NEAT1 provides a scaffold for the key paraspeckle proteins PSP and P54, whereas MALAT1 is an accessory component to these nuclear structures. Chromatin-associated RNA sequencing (CHAR-seq) [30] data shows that XIST was restricted to the X-chromosome, whilst MALAT1 interacted with multiple sites across the genome. However it is not clear what characteristics of MALAT1 endow it with its special properties. Nonetheless, there were very few other RNA species that appeared to globally interact with chromatin, suggesting that this is not a general phenomenon in the nucleus, and that there are very few RNAs that interact in trans.

## Effects of RNA on chromatin structures

At the chromatin fibre level, RNA has been suggested to alter chromatin structure. One proposed mechanism is that RNA can compete with linker histones [42], or neutralise the positively charged tails of histones [43], effectively opening the chromatin fibre. Alternatively, RNAs could recruit [40] or displace [44] structural proteins, transcription factors [45,46] and enzymes that can modify epigenetic marks. Two important chromatin proteins, PRC2 and CTCF, both interact with DNA and chromatin, but also potentially with RNA. For example, *in vitro*, the affinity for G4 RNA by PRC2 is on the order of 25 nM [44], suggesting that in cells, G-tract RNA has the capacity to displace the PRC2 complex, leading to H3K27me3 depletion [44]. But perhaps one of the most contentious issues is whether polycomb is an RNA-binding protein complex [47—49], with arguments both for and against. Similarly, CTCF, a key chromatin architectural protein, has been suggested to bind RNA, which can influence CTCF recruitment and thus alter chromatin loop formation [50,51]. However, there is also conflicting biophysical data suggesting that PRC2 and CTCF may not bind RNA directly in cells [52].

A major reason for discrepancies in these findings may be whether these RNA-protein interactions are “specific” (high affinity) or “non-specific” (low affinity). Using stringent biochemical approaches―such as covalent linkage and affinity purification (CLAP)― polycomb and CTCF do not appear to bind RNA directly [52]. In contrast, methods such as cross-linking and immunoprecipitation (CLIP) suggest that many chromatin proteins do associate with RNA, albeit possibly through weaker or transient interactions [12]. It is therefore feasible that CLAP identifies only high-affinity RNA interactors, while CLIP captures weaker, more transient associations. Within the nuclear environment, where RNA concentrations can be extremely high in specific genomic regions (such as at promoters), mass action could drive RNA-protein interactions [47,50] or displace protein binding [44].

## Formation of a nuclear RNA/protein gel

**Figure 3:**
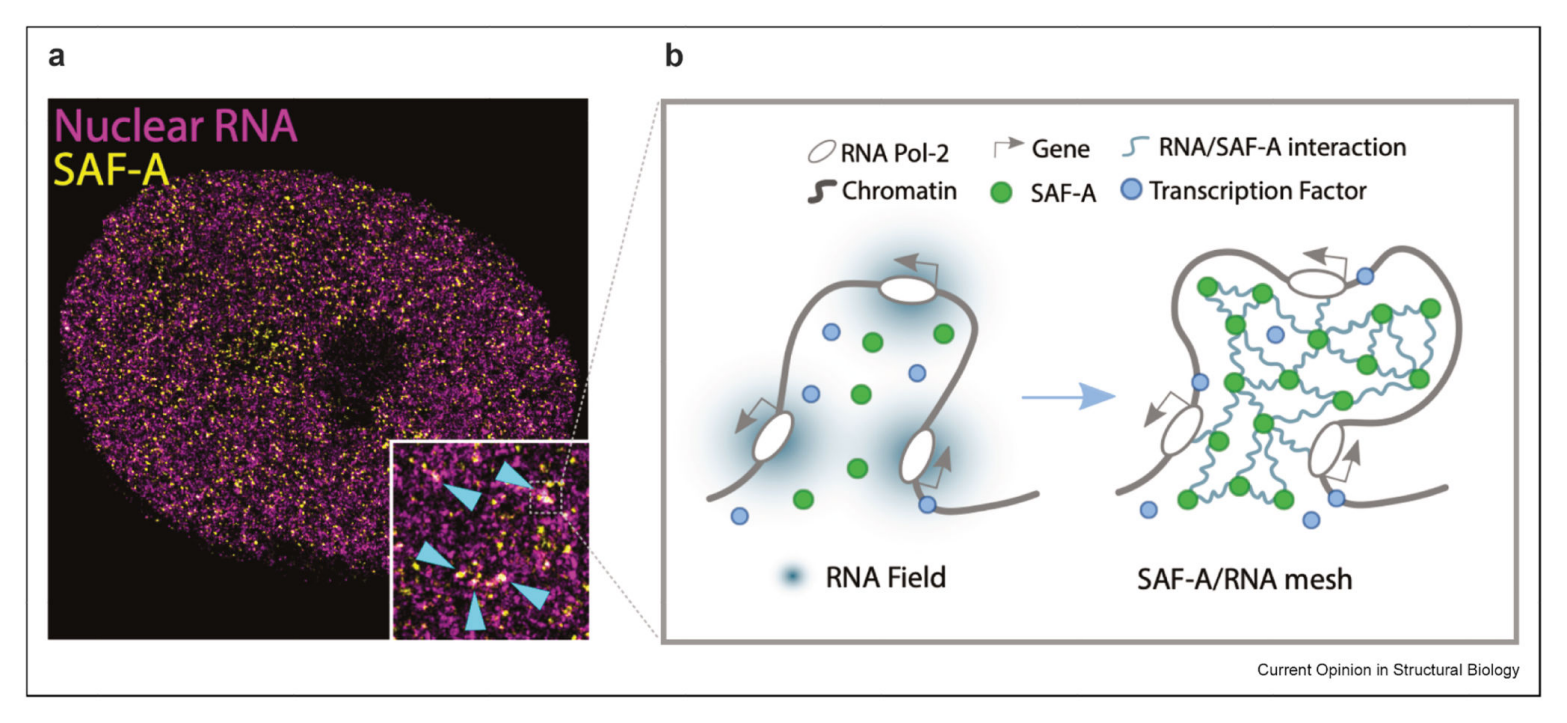
RNA-DNA binding proteins control chromatin structure. **(a)**. STORM microscopy image showing SAF-A protein and labelled RNA species forming an interconnected network of clusters. **(b)**. Cartoon depicting the mechanism of chromatin decompaction dependent on the affinity of SAF-A to bind RNA. We propose that the RNA and SAF-A mesh form a dynamic, interconnected microgel, dependent on nascent RNA transcription.

Over many years evidence has accumulated that suggests RNA can directly impact chromosome structure. Inhibition of transcription compacts large-scale chromatin structures [53], and this appears to be linked to the production of different RNA species in the nucleus, including C_0_T1 repetitive DNA species enriched in LINE and SINE RNAs [27]. Also, other RNA species, that we collectively term RNA debris [5], accumulate and interact to form nuclear structures through interactions with RNA-binding proteins [22,54]. Numerous RNA-binding proteins are likely to form these structures, but one of the best characterised is SAF-A or HNRNPU. SAF-A appears to have multiple roles in the nucleus [55], from regulating chromatin structure [28], XIST inactivation [56], to splicing [57,58]. Back in the 1980s, when researchers started to study mammalian nuclei in detail, they often extracted the protein and DNA, leaving an RNA-rich filamentous structure termed the nuclear matrix [59]. Examination of these samples by electron microscopy revealed an intricate structure that appeared as a framework for organising chromatin [60]. However, the term nuclear matrix belies the dynamic nature of many nuclear proteins and interactions [61], so we have started to reinvestigate these structures using super resolution microscopy combined with biochemistry. We suggest that the RNA/protein mesh-like structure has gel-like properties [62] and can influence the local microenvironment or viscosity [22]. This macromolecular structure spans the nucleus and, we suspect, provides a platform for other nuclear processes including splicing, RNA export and possibly facilitating transcription factors recruitment and retention. The rationale being that a sticky gel-like micro-environment will create individual microdomains analogous to transcription factories [63] that can trap transcriptional regulators. But how can this affect chromatin compaction (Figure 3)? Within the nuclear environment, chromatin fibres interact through a process of bridging-induced phase separation [64], mediated by chromatin-binding proteins [65], and from a thermodynamic perspective, chromatin has a tendency to collapse into a structure that is more energetically stable [66,67]. However, this is influenced by the environment around the chromatin fibre, so the formation of a local RNA/protein gel can both counteract the tendency for the chromatin fibre to collapse, and also stiffen the fibre reducing the likelihood of fibre—fibre interactions through bridging-induced phase separation, which can be observed through biophysical simulations of chromatin folding [68]. In one surprising study, long, highly expressed genes form extended chromatin structures in the nucleus [69]. Reconciling the behaviour of these long genes with data from more typical genes is difficult [66,70], but in this special case nascent RNAs that appear to coat the chromatin fibre might alter the chromatin persistence length, causing the chromatin to expand. Recapitulating these observations using computer simulations has so far been challenging, suggesting that models need to include additional RNA components [62] to influence the nuclear microenvironment [22].

How are RNAs turned over in the nucleus? Surprisingly, only approximately 5% of RNA transcribed in the nucleus is transported to the cytoplasm, leaving a large amount to transiently accumulate, mediate RNA—protein interactions, and be degraded [5,71]. To better understand the half-life of RNA in the nucleus, we undertook pulse-labelling experiments. These suggested that nuclear RNA accumulated for about 30 min to 1 h before being degraded [22]. There are likely to be differences depending on the RNA species [26], but a surprising result is that RNA turnover appeared to be XRN2 dependent [72]. XRN2 is a 5′ to 3′ exonuclease that was first implicated in the process of transcription termination, called the torpedo model [73,74]. In this model XRN2 binds to the 5’ end of RNA transcripts that have not been correctly terminated. The enzyme then degrades the free RNA, eventually meeting the RNA polymerase and displacing it from the DNA template. We speculate that, in addition to the torpedo function of XRN2, it uses its highly processive nuclease activity to degrade nuclear RNA. Consistently, depletion of XRN2 caused an accumulation of nuclear RNA, and a concomitant alteration in the nuclear milieu, effecting local viscosity and protein mobility [22].

## Outlook

**Figure 4:**
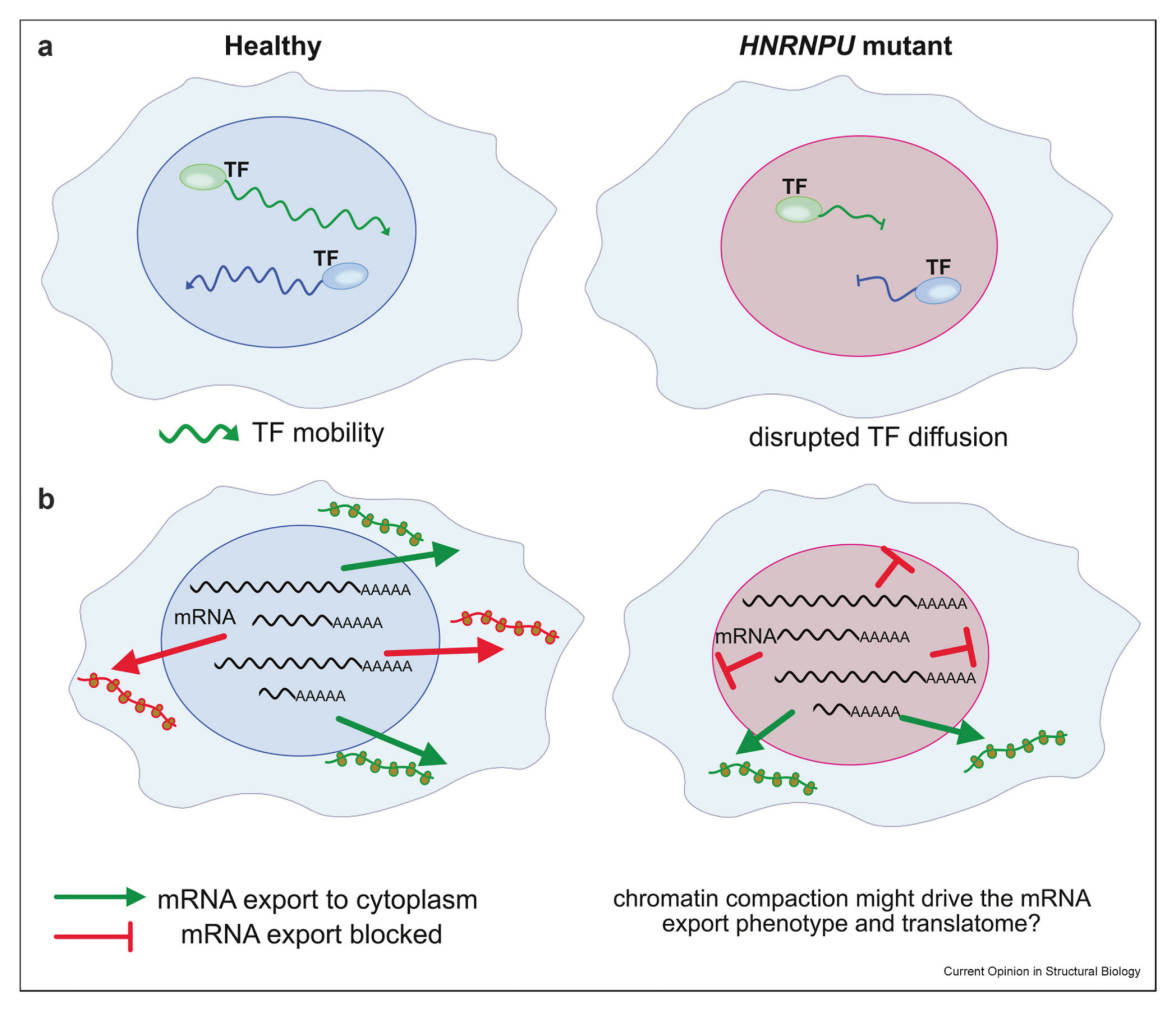
Hypothetical mechanisms of chromatinopathies caused by SAF-A/HNRNPU mutations. (**a)**. Changes in the state of the nucleoplasm and chromatin compaction might drive the disrupted transcription factors (TF) diffusion and altered gene expression. **(b)**. Similarly, mRNA export might be stalled in SAF-A/HNRNPU-mediated disease due to increased chromatin compaction, leading to changes in the translatome.

As RNA is abundant in the nucleus, it is not surprising it has also evolved to play a role in regulating chromatin structure and genome folding. However, this is not easy to unpick as RNA can interact with many different proteins, making it hard to discriminate between specific and non-specific interactions, both of which might be important for nuclear function. Some proteins do have sequence-specific RNA-binding sites, but many others interact non-specifically with RNA, taking advantage of the fact that RNA is a negatively charged polymer [52]. Accumulating data suggests that as RNA can regulate genome folding, there might be transcriptional feedback between transcription and chromatin structure. If this is correct, it will be important to understand how mutations in structural nuclear proteins influence gene regulation and, in some cases cause human diseases [55,75]. When we and others first started working on SAF-A [27,28], the main interest was in its role in regulating large-scale chromatin structures [76]. However, numerous recent sequencing projects have found that large numbers of patients with uncharacterised disorders have mutations in chromatin proteins including SAF-A. Patients with mutations in SAF-A exhibit a wide spectrum of neurodevelopmental disorders, with evidence of epilepsy and autism-like symptoms [75,77]. The molecular basis for these diseases are not well characterised [55,78—80], but potentially, altering the nuclear environment can influence RNA processing and export (Figure 4), opening up exciting new studies that will shed further light on the role of RNA in genome folding.

## Data Availability

No data was used for the research described in the article.
